# Microglial clock dysfunction during neuroinflammation impairs oligodendrocyte progenitor cell recruitment and disrupts neuroimmune homeostasis

**DOI:** 10.3389/fimmu.2025.1620343

**Published:** 2025-07-07

**Authors:** Qingqing Lu, Jin Young Kim

**Affiliations:** ^1^ Department of Biomedical Sciences, College of Biomedicine, City University of Hong Kong, Hong Kong, Hong Kong SAR, China; ^2^ Tung Biomedical Sciences Centre, City University of Hong Kong, Hong Kong, Hong Kong SAR, China

**Keywords:** neuroinflammation, microglia, circadian clocks, microglial homeostasis, oligodendrocyte progenitor cells, glial-glial communication

## Abstract

**Introduction:**

Circadian clocks generate daily physiological rhythms and regulate immune functions, including cytokine production and inflammatory responses. Although time-of-day–dependent variation in microglial immune activity has been reported, how intrinsic microglial clocks respond to neuroinflammatory stimuli and influence microglial function remains unclear.

**Methods:**

We induced neuroinflammation via intraperitoneal injection of lipopolysaccharide (LPS) and isolated microglia from control and LPS-treated mouse brains. To examine circadian clock dynamics and downstream targets, we performed time-series gene expression analyses. To assess the functional relevance of microglial clocks, we transplanted either wild-type or *Bmal1*-deleted microglia, as well as control or neuroinflammatory microglia, into the corpus callosum of NG2^DsRed^ reporter mice and evaluated oligodendrocyte progenitor cell (OPC) recruitment.

**Results:**

LPS-induced neuroinflammation triggered a phase shift in the core clock gene *Bmal1* and disrupted the rhythmic expression of its targets, including *Per1, Iba1, Itgam*, and *Ccl5*, resulting in sustained microglial activation. Transplanted wild-type microglia effectively recruited OPCs, whereas both *Bmal1*-deleted and neuroinflammatory microglia failed to recruit OPCs, indicating that disrupted microglial clock function promotes persistent activation and impairs glial–glial communication.

**Discussion:**

These findings identify microglial circadian clocks as key regulators of homeostatic function and glial–glial communication. Preserving intrinsic clock function in microglia may represent a strategy to mitigate neuroinflammatory damage and support white matter integrity.

## Introduction

Microglia, the resident immune cells of the central nervous system, constitute approximately 10-15% of total glial cells and play critical roles in maintaining brain homeostasis through immune surveillance, synaptic remodeling, and defense against pathogens (1–3). In their surveillant (resting) state, microglia exhibit highly branched morphologies with dynamic and motile processes that continuously monitor the surrounding brain environment (4–10). Recent evidence shows that these motile processes not only reflect microglial vigilance but also contribute to synaptic remodeling and intercellular communication (7, 11–15). These microglial morphology and behavior display diurnal variation, suggesting regulation by circadian clocks.

While molecular circadian clocks in neurons and astrocytes have been extensively studied (16, 17), research into microglial clocks has remained relatively limited. However, emerging evidence indicates that microglia contain functional circadian machinery—endogenous oscillators that drive circadian rhythms in most cell types (18). Core clock components such as brain and muscle Arnt-like protein 1 (*Bmal1*), period 1/2 (*Per1/2*), and cryptochrome 1/2 (*Cry1/2*) are rhythmically expressed in microglia (19–22). The circadian transcription factors BMAL1 and circadian locomotor output cycles kaput (CLOCK) form a heterodimer that initiates transcription of their own inhibitors, *Per1/2* and *Cry1/2* (23–26). PER and CRY proteins subsequently form large inhibitory complexes that suppress BMAL1/CLOCK activity, forming a transcription nal-translational negative feedback loop with an approximately 24-hour periodicity (27, 28). This molecular oscillator also regulates thousands of circadian output genes controlling both general and cell-type-specific physiological functions (29–31). This suggests that microglial clocks may orchestrate diverse microglial processes across the day.

Microglia rapidly respond to environmental stimuli such as injury or infection by transitioning from resting to activated states (32). Activated microglia are characterized by retracted processes, amoeboid morphology, increased ionized calcium-binding adaptor molecule 1 (IBA1) and CD11b (also known as integrin alpha M, ITGAM) levels, and enhanced production of pro-inflammatory cytokines and chemokines (33–35). Recent studies suggest that microglial immune competence is under circadian regulation. Time-of-day-dependent variation in responses to inflammatory stimuli, such as neuronal injury or lipopolysaccharide (LPS) exposure, have been consistently reported (36). For example, interleukin-1β (IL-1β) and tumor necrosis factor-α (TNF-α) responses are enhanced when microglia are challenged during the active phase of the circadian cycle in rodents (9, 20, 34). These observations suggest that microglial sensitivity and responsiveness to inflammatory stimuli and immune function are modulated by intrinsic circadian clocks.

Circadian disruption has been increasingly implicated in neurological diseases, including neurodegenerative and demyelinating disorders (16, 37). Since microglia are central players in both homeostatic maintenance and immune responses in the brain (22, 38), disrupted microglial clocks may contribute to disease progression by impairing physiological functions such as glial support and repair. While *Bmal1* deletion in microglia has been shown to impair rhythmic cytokine expression (22, 39), it remains unclear how microglial clocks are altered during an inflammatory response, and whether these alterations affect the non-immune functions they normally perform under physiological conditions.

Here, we investigate whether microglial clocks regulate a non-immune homeostatic function—oligodendrocyte progenitor cell (OPC) recruitment—which is essential for white matter maintenance and myelin repair. Using *in vitro* and *in vivo* approaches, we show that microglia contain self-sustained clocks that generate circadian output rhythms under physiological conditions. We further demonstrate that intact microglial clocks are required for OPC recruitment in the healthy brain. Once neuroinflammation is induced, microglial clocks become phase-shifted or disrupted, which is accompanied by a loss of OPC recruitment and a shift toward sustained inflammatory activation. Together, these findings identify microglial clocks as regulators of supportive homeostatic function and reveal how clock disruption contributes to a functional transition from homeostatic to inflammatory microglial states.

## Materials and methods

### Mice

All mouse lines were maintained in Laboratory Animal Research Unit, City University of Hong Kong. mPer2::Luc (RRID: IMSR_JAX:006852, B6.129S6-Per2tm1Jt/J) (40); CX3CR-1^GFP^ (RRID: IMSR_JAX:005582, B6.129P2(Cg)-Cx3cr1^tm1Litt^/J) (41); NG2^DsRed^ (RRID:I MSR_JAX:008241, STOCK Tg(Cspg4-DsRed.T1)1Akik/J) (42); Aldh1l1-EGFP/Rpl10a (RRID: IMSR_JAX:030248, B6; FVB-Tg(Aldh1l1-EGFP/Rpl10a) JD133Htz/J) (43) and *Bmal* knockout mice (RRID: IMSR_JAX:009100, B6.129-Arntl^tm1Bra^/J) (44) were purchased from Jackson Laboratories. All mice were maintained in a 12:12h light-dark cycle at 20-24°C with 50%–70% humidity. All mouse experiments were performed in accordance with the protocol approved by the Institutional Animal Research Ethics Sub-Committee of City University of Hong Kong and Department of Health, The Government of The Hong Kong Special Administrative Region.

### Primary microglia cultures

Primary microglia were cultured from cortex of the postnatal day 0-2 (P0–2) pups. After decapitation, craniotomy and meninges removal in dissection medium [1x HBSS (Gibco) + 2.5 mM HEPES (Gibco) + 5.4 g/L glucose (Sigma-Aldrich) + 100 units/mL penicillin/100 μg/mL streptomycin (Gibco)], dissected brains from postnatal day 0-2 (P0-P2) C57BL/6J mice were digested. The tissue was triturated after trypsin neutralization, filtered through a 70 μm cell strainer (Falcon), and seeded in 0.1 mg/mL Poly-D-Lysine (PDL)-coated flasks. Cultures were maintained at 37°C with 5% CO&_2_, with medium changed every 3–4 days. Mixed glial cells were ready for subculture or experiments after fully confluence for experimentation at 6–8 days *in vitro*.

Mixed glia cultures were digested with trypsin. After trypsinization determined by culture medium [1x HBSS (Gibco) + 2.5mM HEPES (Gibco) + 5.4g/L glucose (Sigma-Aldrich) + 100 units/mL penicillin + 100 μg/mL streptomycin (Gibco)], microglia were purified by cluster of differentiation molecule 11B (CD11b) beads (#130-097-142, Miltenyi) and followed the magnetic-activated cell sorting (MACS) protocol according to Miltenyi’s instructions. After elution and centrifugation, microglia were resuspended with culture medium and plated in a dish pre-coated by Poly-D-Lysine.

### Bioluminescence assay

Primary microglia were cultured from the cortex of P0–2 pups of the mPer2::Luc mouse line, which expresses a PER2::LUCIFERASE reporter in various tissues for around 1 week until full growth, then purified and cultured for 3 days. To synchronize all the cells in the same plate, microglia were treated with 100 μM Dexamethasone (Cat# D4902, Sigma) in Lumicycle Medium [1% DMEM (Gibco) + 0.348% Glucose (Sigma-Aldrich) + 10 mM HEPES (Gibco) + 10% Horse serum (Gibco) + 350 mg/L Bicarbonate solution (Gibco) + 140 U/mL Penicillin-Streptomycin (Gibco)] for 2 h. After synchronization, culture media was changed by Lumicycle medium with 100 μM D-Luciferin (Cat# E1602, Promega) as the substrate of luciferase. The culture plate was sealed with a glass coverslip by vacuum grease and kept in the LumiCycle machine (Actimetrics) placed in the cell incubator at 37°C with 5% CO_2_. Bioluminescence (counts/sec) produced by luciferase from each plate was recorded and the oscillation curve was calculated by the software Lumicycle Analysis (45).

### Immunohistochemistry

8- to 12-week-old adult mice were perfused with 4% (wt/vol) paraformaldehyde (PFA) in 1x phosphate-buffered saline (PBS) at circadian time (CT) 06 and their brains were dehydrated in 30% (wt/vol) sucrose (ChemCruz). Fixed coronal sections with 20μm thickness in proper position were attached to adhesion slides and blocked with PGBA blocking buffer containing 0.1% gelatin (Sigma-Aldrich), 1% BSA (Sigma-Aldrich), 0.002% sodium azide (Sigma-Aldrich), 10% normal goat serum (Jackson Immuno Research), and 0.5% Triton X-100 (Sigma-Aldrich) in 0.1 M phosphate buffer [PB, 71.7 mM K_2_HPO_4_ (Sigma-Aldrich) + 28.3 mM KH_2_PO_4_ (Sigma-Aldrich)] for 1 hour under room temperature. Then, the sections were incubated with primary antibodies in blocking buffer overnight at 4°C. After washing for three times with 0.1M PB, each time 10 minutes, brain sections were incubated with secondary antibodies in blocking buffer for 1 hour under room temperature without light. Again, three times of wash with PB were performed to remove secondary antibodies. Finally, samples were mounted with mounting medium containing 4’,6-diamidino-2-phenylindole (DAPI; Vetashield, H-1200).

The following antibodies were used in this study for IHC: IBA1 (#019-19741, Wako), 1:500; CD11b (#101202, Biolegend), 1:100; S100β (ab52642, Abcam), 1:100; GFAP (#835301, Biolegend), 1:1000; NG2 (#AB5320, Merck), 1:200; Ki67 (#14-5698-80, Invitrogen), 1:500; Alexa Fluor 488 donkey anti-mouse, Alexa Fluor 594-donkey anti-mouse, Alexa Fluor 647-donkey anti-mouse, Alexa Fluor 488-donkey anti-rabbit, Alexa Fluor 594-donkey anti-rabbit, Alexa Fluor 594-donkey anti-rat, Alexa Fluor 647-donkey anti-rat antibody, 1:400 (all from Jackson Immuno Research).

### Imaging

Fluorescent images were obtained using A1R HD25 Confocal Microscope (Nikon) by NIS-Elements imaging software (Nikon) (46). Images were taken with 20x objective lens and magnification of 1024*1024. Images with Z stacks had 5 stacks and the step between each stack was 2 μm. When making Z stacks with max intensity projection, 3 stacks with the highest and best signal intensities were used for the morphology analysis or cell count in the injection site.

### LPS-induced neuroinflammatory mouse model

Lipopolysaccharides (LPS, L7895, Sigma) was dissolved in endotoxin-free DPBS (#A1285601, Gibco) to make the 1 mg/mL stock for the injection. To induce the neuroinflammatory reaction in mouse brain, 5 mg/kg LPS was injected intraperitoneally (i.p.) to 8- to 12-week-old adult mice. To maintain the consistent circadian times across animals, LPS injection was performed at CT04. After specific days of injection, mice were sacrificed and their brains were harvested for the following experiments.

### Microglia isolation

Brains were harvested at CT04 from 8- to 12-week-old adult mice, and dissected cortices were digested in trypsin. The pellet was triturated slowly and gently in 4 mL culture medium, and then transfer cell suspension to 70 μm cell strainer and washed by another 1 mL culture medium. The cells were pelleted and resuspended in 2 mL brain red blood cell lysis buffer [155 mM NH_4_Cl (Sigma-Aldrich) + 12 mM NaHCO_3_ (Sigma-Aldrich) + 0.1 mM EDTA (Sigma-Aldrich)] and incubated for 5 minutes at room temperature. Cells were washed once with MACS buffer [0.5% w/v Bovine Serum Albumin (BSA; Sigma-Aldrich) + 2 mM Ethylenediaminetetraacetic Acid (EDTA; Affymetrix) in phosphate buffered saline [PBS; 137 mM NaCl (Affymetrix) + 2.7 mM KCl (Sigma-Aldrich) + 8 mM Na_2_HPO_4_ (Sigma-Aldrich) + 1.5 mM KH_2_PO_4_ (Sigma-Aldrich)] to remove red blood cell lysis buffer thoroughly. Then, single cells were resuspended in MACS buffer.

To purify glia cells and remove neurons and oligodendrocytes from the mixed single cells, 60 μL myelin removal beads (#130-096-731, Miltenyi) were added to the cell suspension and purified by MACS according to Miltenyi’s instructions. To purify microglia from the mix glia suspends, 10 μL CD11b beads (#130-097-142, Miltenyi) were added to the myelin-removed cell suspension purified by MACS according to Miltenyi’s instructions. Countess II Automated Cell Counter (AMQAX1000, Invitrogen) was used to determine cell number with 0.4% of Trypan blue staining 0.4% (2295044, Invitrogen) for live cell concentration.

### RNA extraction and quantitative real-time polymerase chain reaction

For circadian gene expression analyses, brains were harvested every 4 hours over 28 hours from 8- to 12-week-old adult mice, providing seven time points that span over a complete circadian cycle under both control and post-LPS conditions. Purified microglia were dissolved in TRIzol (15596026, Invitrogen) and then kept in -80°C for the RNA extraction. Total RNA was extracted following manufacturer’s instruction by Invitrogen. Nanodrop spectrophotometry was used to assess RNA purity and concentration and 400 ng of total RNA was used for the following steps. Genomic DNA removal and RNA reverse transcription were performed by PrimeScript RT Reagent Kit with gDNA Eraser (Perfect Real Time) (TaKaRa). qRT-PCR with cDNA products were performed with SYBR Premix Ex Taq (Tli RNase H Plus) (TaKaRa) in Applied Biosystems QuantStudioTM 3 Real-Time Polymerase Chain Reaction System. Primers used for the qRT-PCR are listed in **Table 1**.

**Table 1:** Primers used for qRT-PCR.

Genes	Forward sequence (from 5’ to 3’)	Reverse sequence (from 5’ to 3’)
*Rpl30*	GCTGGAGTCGATCAACTCTAGG	CCAATTTCGCTTTGCCTTGTC
*Rps13*	TCCCTCCCAGATAGGTGTAATCC	TCCTTTCTGTTCCTCTCAAGGT
*Bmal1*	TGACCCTCATGGAAGGTTAGAA	GGACATTGCATTGCATGTTGG
*Per1*	CAGCTGGGCCGGTTTTG	CACTTTATGGCGACCCAACA
*Iba1*	CTTTTGGACTGCTGAAGGC	GTTTCTCCAGCATTCGCTTC
*Itgam*	ATGGACGCTGATGGCAATACC	TCCCCATTCACGTCTCCCA
*Il-1β*	GCAACTGTTCCTGAACTCAACT	ATCTTTTGGGGTCCGTCCAACT
*Ccl3*	TTCTCTGTACCATGACACTCTGC	CGTGGAATCTTCCGGCTGTAG
*Ccl5*	AGATCTCTGCAGCTGCCCTCA	GGAGCACTTGCTGCTGGTGTAG
*Ccl12*	CAGTCCTCAGGTATTGGCTGG	GGGTCAGCACAGATCTCCTT

### Cytokine screening

A total of 1x10^7^ cells purified from cortices harvested at CT04 within 2 to 3 adult mice (8- to 12-week-old adult mice) were lysed by 1 mL lysate buffer [1% Igepal (CA-630, Sigma-Aldrich), 20 mM Tris-HCl (pH 8.0) (Affymetrix), 137 mM NaCl (Affymetrix), 2 mM EDTA (Affymetrix), 200 mM Sodium Orthovanadate (Sigma-Aldrich), 5 mM NaF (Sigma-Aldrich) with freshly added cOmplete^™^ protease inhibitor (Roche)] with gently rock at 4°C for 30 minutes. After incubation, cell suspension was collected into a clean tube by centrifugation. Protein concentration was measured by using Braford assay (Bio-rad). Cytokine screening was performed by using Mouse Cytokine Antibody Array, Panel A (ARY006, R&D). Bio-Rad ChemiDoc (Bio-rad) was used to take the images. Image quantification was performed with ImageJ measurement.

### Microglia transplantation

To transplant microglia into the corpus callosum (CC) of recipient mouse brain, 8- to 12-week-old adult mice were narcotized with 200 μl Ketamine (final concentration: 1%; #HK-37715, Alfasan)/Xylazine (final concentration: 0.1%; #HK-56179, Alfasan) in filtered saline (0.9% NaCl in water). Digital Mouse Stereotaxic Instruments (Stoelting) was used to fix the mice head and the skull was exposed by vertical cut on the head skin. After verifying skull flatness by moving the injector with glass needle filled with mineral oil (M8410-100ML, Sigma-Aldrich), the injection coordinates relative to the bregma in mm were used to reach the CC, AP: -1.70, ML: ± 1, DV: 0.25, and 1x10^5^ microglia in total volume of 1 μL were injected. with Nanoject III Programmable Microinjector and Wiretrol™ I glass micropipette (Drummond) at a rate of 13 nl/s in each side. Pipettes remained post-injection for 5 minutes to prevent reflux. The wound on the scalp was sealed with Vetbond surgical glue (3M), followed by postoperative monitoring until recovery (47). Microglia for injection were purified at CT04, corresponding to the circadian activation phase, and transplanted into the CC at CT10, based on the time required for microglia isolation and preparation. Brains were collected three days post-transplantation, allowing the transplanted microglia to remain in the CC for approximately three full circadian cycles.

### Statistical power calculation

To evaluate whether our sample size was sufficient to detect group differences, we calculated statistical power based on the observed effect size. Assuming a two-tailed Student’s t-test, α = 0.05, and a large expected effect size (Cohen’s d = 1.5), the statistical power was approximately 65% for comparisons with n = at least 3 per condition. This effect size was based on consistent and robust differences observed in microglial branch number, marker intensity, and OPC recruitment. Although the sample size is modest, statistically significant differences were observed across multiple independent experiments. The combination of reproducible findings and large effect size supports the biological relevance and reliability of our conclusions. A Cohen’s d of 1.5 is considered a very large effect, and under such conditions, statistical significance can be reasonably detected with limited replication. While the power is below the conventional 80% threshold, we interpret these results as meaningful, and future studies with larger cohorts will further validate and extend these findings.

### Quantification and statistical analysis

Data were presented as mean ± standard deviation (SD). Prior to applying significance analysis, for data sets with N > 20, the assumption of normality for continuous variables was evaluated by quantile-quantile (Q-Q) plots. Departures from the straight diagonal reference line were interpreted as evidence of non-normality. When the number of biological replicates was fewer than seven (N < 7), normality testing was not conducted, as standard normality tests are not reliable with small sample sizes. Instead, all individual data points are presented in each graph to allow a transparent assessment of data distribution. Difference comparisons between two different conditions were carried out using an unpaired two-tailed Student’s t-test. All images were created and analyzed by NIS-Elements BR 5.21.00 and quantified using ImageJ. All statistical analyses were performed using GraphPad Prism 9 (San Diego, CA). In all cases, results were considered statistically significant at *P* < 0.05.

Statistical analysis of circadian rhythmicity of all examined genes was determined by CircaCompare (48) or MetaCycle (49).

## Results

### Microglia exhibit self-sustained circadian rhythms

**Figure 1:**
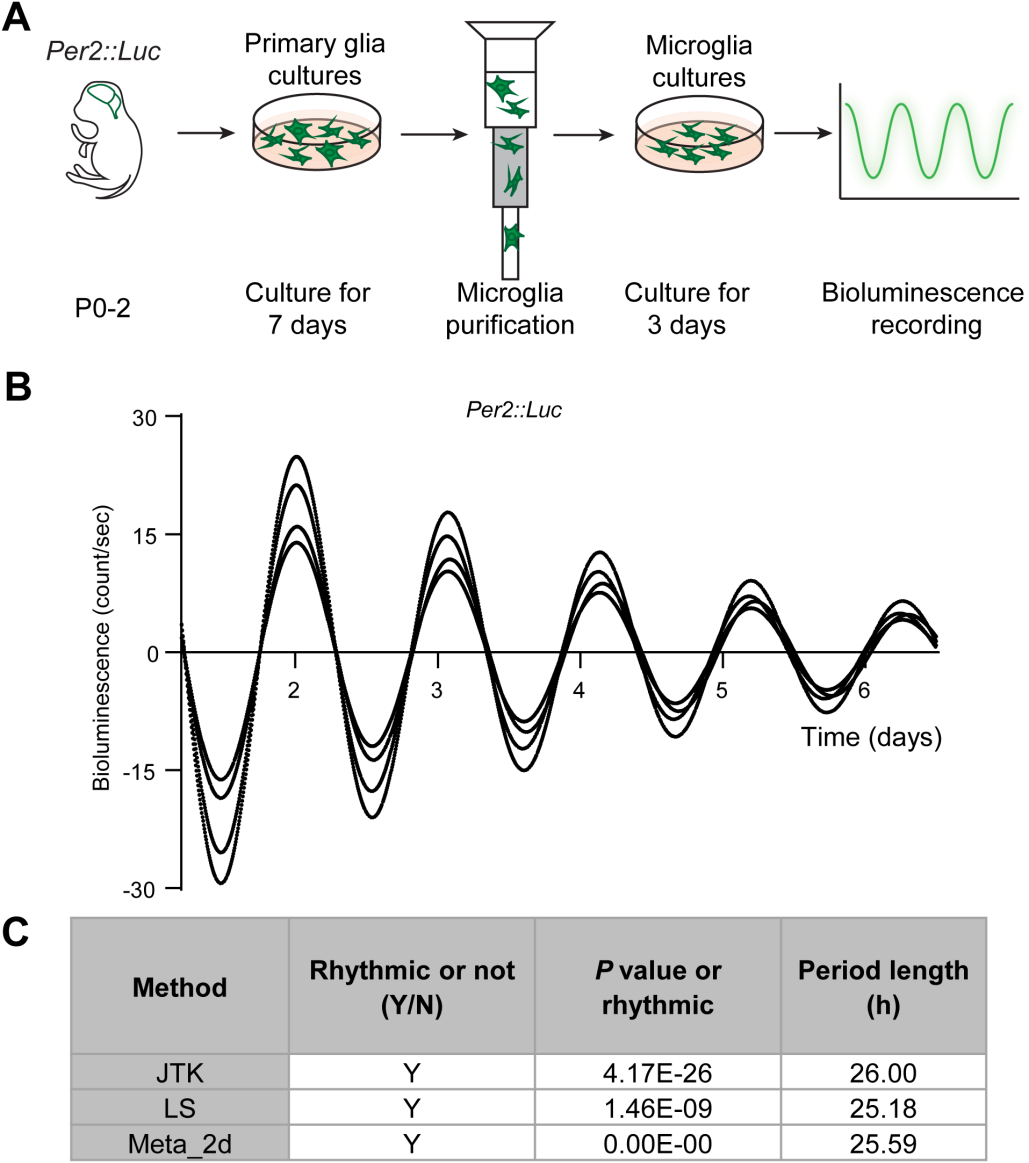
Microglia exhibit self-sustained circadian rhythms. **(A)** Experimental workflow of the bioluminescence assay. Luciferase-conjugated PER2 levels were recorded in microglial cultures. **(B)** Circadian oscillations of PER2 are shown as bioluminescence traces over six consecutive days. X- and Y-axis represent recording time (days) and bioluminescence counts per second, respectively. Traces are aligned to day 1. Representative of four independent experiments. **(C)** Statistical analysis of circadian rhythmicity performed using the R package MetaCycle, which integrates three independent methods: JTK_CYCLE (JTK), Lomb-Scargle (LS), and ARER (Meta_2d). Trace is considered rhythmic when the *P* value is < 0.05. Y, yes, rhythmic; N, no, non-rhythmic. Period length in hour (h).

Most cell types, including microglia, contain endogenous circadian clocks. However, the robustness of rhythmicity can vary across cell types and may be reinforced by interactions with neighboring cells (50, 51). To determine whether microglial clocks are self-sustained without external inputs, we cultured cortical microglia from mPer2::Luciferase knock-in mice (mPer2::Luc) (52) and monitored real-time bioluminescence for six consecutive days (**Figure 1A**). The luciferase recordings revealed sustained oscillatory rhythms with a period of 25–26 hours (**Figures 1B, C**). This confirms that microglia contain endogenous molecular clocks capable of generating self-sustained circadian rhythms independently of external inputs.

### Microglial activation states oscillate across the circadian cycle under physiological conditions

**Figure 2:**
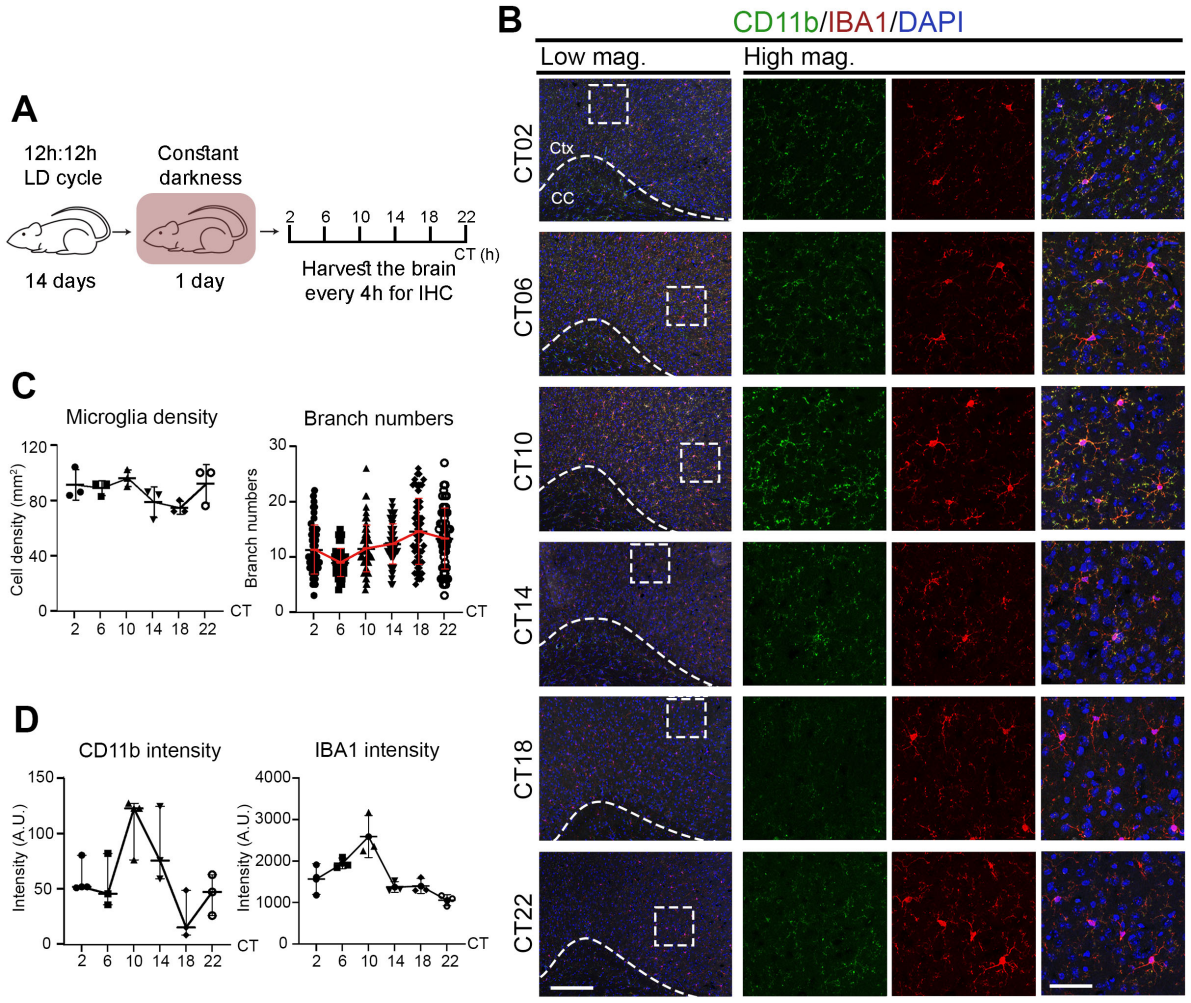
Microglial activation states oscillate across the circadian cycle under physiological conditions. **(A)** Experimental workflow. Mice were entrained under 12h:12h light:dark (LD) cycle for 14 days, followed by 1 day in constant darkness (DD). Brains were harvested every 4 hours across the circadian cycle for immunochemistry (IHC). **(B)** Confocal images of brain sections stained for CD11b (green) and IBA1 (red) at various circadian times (CT, hours). DAPI (blue) serves as a nuclear counterstain. Dashed boxes in lower magnification (Low mag.) images indicate regions enlarged in higher magnification (High mag.) panels. Dashed line outlines the cortex (Ctx) from the corpus callosum (CC). Images are shown as maximum intensity projections of three z-stack layers. Scale bars (white bars in bottom panels): 200 μm (Low mag.), 50 μm (High mag.). **(C, D)** Quantification of **(B)**, shown as mean ± SD. **(C)** Microglial density (IBA1&^+^ cells/mm²) and branch number per IBA1&^+^ microglia. n = 3 mice per CT. Each dot represents one mouse (density) or one cell (branching; ≥56 cells per CT from 3 mice). **(D)** Mean intensity of IBA1 and CD11b at each CT. Each dot represents one mouse. n = 3 mice per CT.

Since microglia closely interact with neurons and glia (oligodendrocytes and astrocytes) in the brain, we next examined their *in vivo* circadian rhythms by monitoring activation states across the day (**Figure 2A**). Immunostaining for IBA1, a pan-microglial marker, revealed no significant change in microglial number across circadian time (CT) points. However, the number of branches per IBA1&^+^ cell oscillated, peaking at CT18 and troughing at CT06 (**Figures 2B, C**; **Table 2**)—reduced branching at CT06 indicating a more activated microglial state. In addition, the intensity of IBA1 and CD11b—both associated with microglial activation—also exhibited rhythmic oscillations, peaking around CT10 (**Figures 2B, D**; **Table 2**). These results indicate that microglial activation states oscillate under physiological conditions, with a higher activity around CT06 and a lower activity around CT18, correlating with circadian rhythmicity.

**Table 2:** Circadian rhythmicity analyses of microglial states under physiological conditions.

Parameter	Method	Rhythmic or not (Y/N)	*P* value or rhythmic	Period length (h)
Microglia density	JTK	N	4.41E-01	NA
	LS	N	5.88E-01	NA
	Meta_2d	N	6.09E-01	NA
Branch numbers	JTK	Y	5.99E-03	20
	LS	N	4.09E-01	NA
	Meta_2d	Y	3.44E-02	20
Intensity of CD11b	JTK	Y	6.38E-39	24
	LS	Y	0.00E+00	20
	Meta_2d	Y	0.00E+00	20
Intensity of IBA1	JTK	Y	1.90E-04	24
	LS	N	7.14E-02	NA
	Meta_2d	Y	1.66E-04	24

Statistical analysis of rhythmicity corresponding to **Figure 2** using the R package MetaCycle, which integrated three independent methods: JTK_CYCLE (JTK), Lomb-Scargle (LS), and ARER (Meta_2d). Rhythmicity is considered significant when the *P* value is < 0.05. Y, yes, rhythmic; N, no, non-rhythmic; NA, not available. Period length in hour (h).

### Microglial activation is further enhanced during neuroinflammation

**Figure 3:**
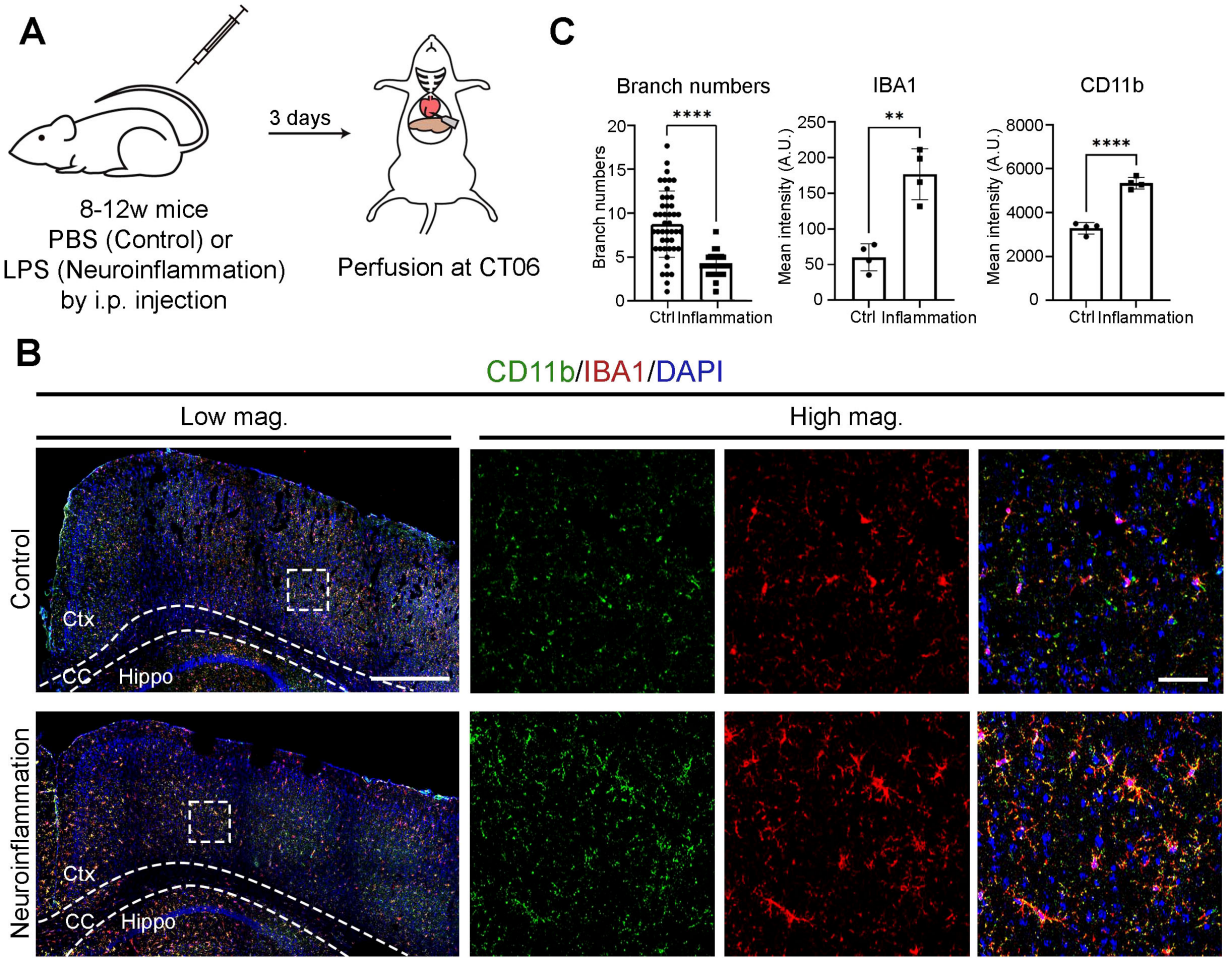
Microglial activation is further enhanced during neuroinflammation. **(A)** Experimental workflow illustrating the induction of neuroinflammation. Mice received an intraperitoneal (i.p.) injection of either PBS (control) or lipopolysaccharide (LPS) at circadian time (CT) 04. Brains were harvested three days post-injection at CT06. **(B)** Confocal images showing CD11b (green) and IBA1 (red) immunostaining. DAPI (blue) serves as a nuclear counterstain. Dashed boxes in lower magnification (Low mag.) images indicate regions enlarged in higher magnification (High mag.) panels. Dashed lines outline the cortex (Ctx), corpus callosum (CC), and hippocampus (Hippo). Images are maximum intensity projections of three confocal z-planes. Scale bars (white bars in top panels): 200 μm (Low mag.), 50 μm (High mag.). **(C)** Quantification of data from **(B)**, presented as mean ± SD. n = 2 mice per condition. Each dot represents one microglial cell (branch number; ≥31 cells per condition from 2 mice) or one hemi-cerebrum (mean intensity). Asterisks indicate statistical significance (Student’s t-test). **P < 0.01; ****P < 0.001.

Microglia transition into activated states in response to a variety of pathological conditions (8, 10). To compare activation states under physiological versus pathological conditions, we induced neuroinflammation through intraperitoneal (i.p.) LPS injection (**Figure 3A**). Immunostaining revealed a progressive increase in IBA1 and CD11b intensity from Day 1 to Day 3 post-injection, with peak expression observed on Day 3 (**Supplementary Figure S1**). At CT06—a time point when microglia are normally most active—neuroinflammatory brains showed microglia with fewer branches and markedly increased IBA1 and CD11b intensity compared to PBS-injected controls (**Figures 3B, C**). These findings indicate that while microglial activation exhibits circadian oscillations under physiological conditions, neuroinflammation leads to a markedly enhanced activation state. This implies a functional shift from homeostatic surveillance to immune response.

### Neuroinflammation alters microglial clock rhythmicity

**Figure 4:**
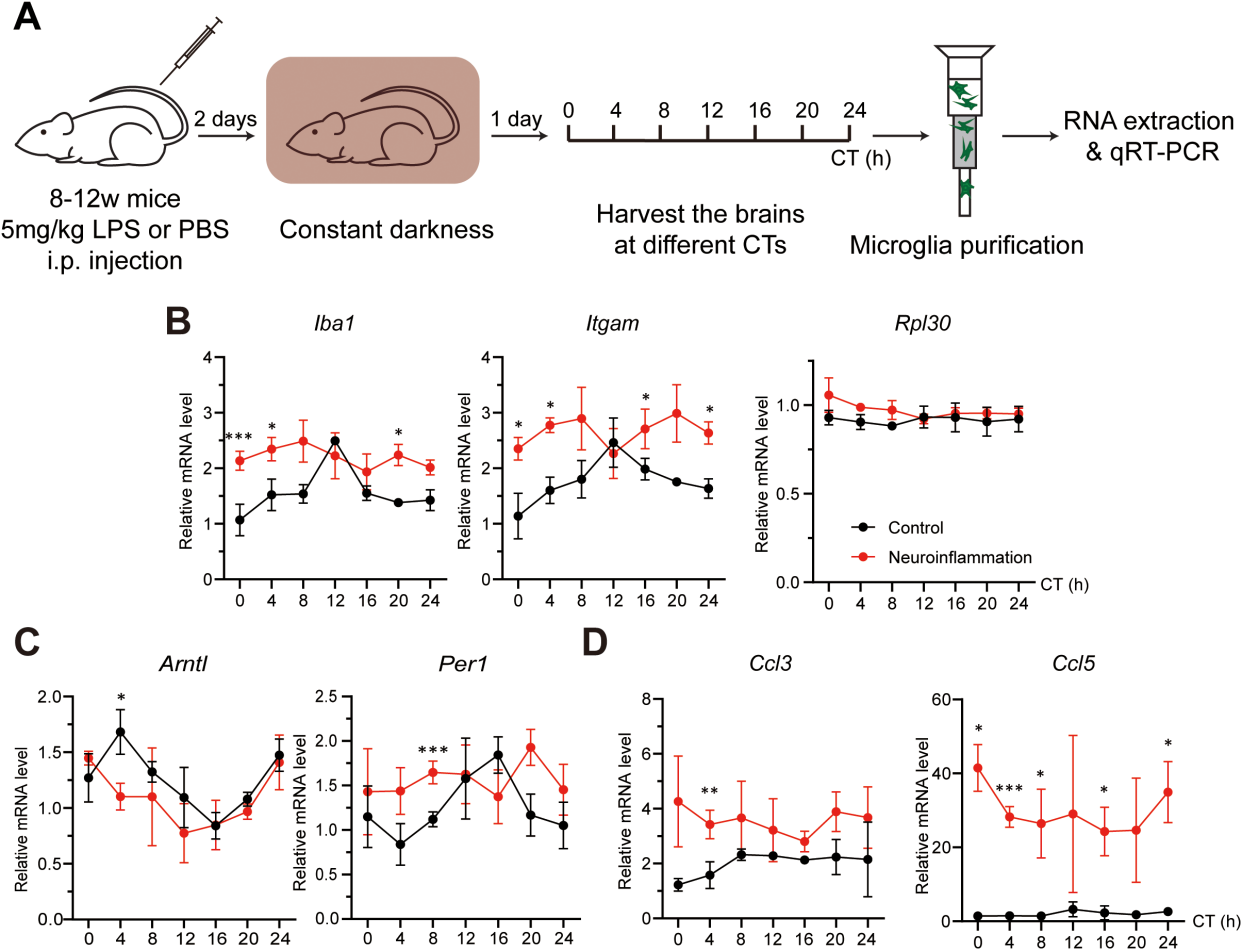
Neuroinflammation alters microglial clock rhythmicity. **(A)** Experimental workflow illustrating the induction of neuroinflammation and preparation of microglia at various circadian times (CTs). Mice received an intraperitoneal (i.p.) injection of either PBS (control) or lipopolysaccharide (LPS, neuroinflammation) at CT04. Brains were harvested every 4 hours across the circadian cycle on day 3 post-injection. **(B-D)** qRT-PCR analysis showing the effects of neuroinflammatory conditions on the expression of **(B)** microglial activation markers (*Iba1* and *Itgam*) and an irrelevant control gene (*Rpl30*), **(C)** core clock genes (*Bmal1* and *Per1*), and **(D)** pro-inflammation chemokines (*Ccl3* and *Ccl5*). Expression values are normalized to a control gene (*Rps13*) and presented as mean ± SD. Black and red lines represent control and neuroinflammatory microglia, respectively. n = 3 mice per CT. Asterisks indicate statistical significance (Student’s t-test). *P value < 0.05; **P value < 0.01; ***P value < 0.005.

Since microglial activation is elevated under neuroinflammatory conditions, we next examined whether this state is associated with changes in microglial clocks. Microglia were purified every 4 hours across a full circadian cycle from both control and neuroinflammatory mice, and gene expression was analyzed (**Figure 4A**). In control microglia, *Iba1* and *Itgam* exhibited circadian oscillations, consistent with IBA1 and CD11b immunostaining results (**Figure 4B**; **Table 3A**). In contrast, neuroinflammatory microglia showed overall induced expression of these genes, with *Iba1* displaying a significant phase shift and *Itgam* losing rhythmicity (**Table 3A**). These results suggest that neuroinflammation disrupts clock-regulated expression patterns of microglial activation genes.

**Table 3:** Circadian rhythmicity analysis of microglial activation markers, core clock components, and chemokines under control and neuroinflammatory conditions.

Gene	Parameter	Rhythmic or not (Y/N)/Value
*Rpl30*	Ctrl is rhythmic.	N
	Neuroinflammation is rhythmic.	N
A. *Iba1*	Ctrl is rhythmic.	Y (2.10E-04)
	Neuroinflammation is rhythmic.	Y (4.57E-02)
	*Significance* in phase difference	Y (0.02)
	Phase difference estimate	4.72
	Ctrl peak time	CT7
	Neuroinflammation peak time	CT12
	*Significance in* amplitude difference	N
A. *Itgam*	Ctrl is rhythmic.	Y (1.97E-04)
	Neuroinflammation is rhythmic.	N
	*Significance* in phase difference	NA
	Phase difference estimate	NA
	Ctrl peak time	CT13
	Neuroinflammation peak time	NA
	*Significance in* amplitude difference	NA
B. *Bmal1*	Ctrl is rhythmic.	Y (8.39E-04)
	Neuroinflammation is rhythmic.	Y (4.75E-06)
	*Significance* in phase difference	Y (3.00E-02)
	Phase difference estimate	-2.71
	Ctrl peak time	CT4
	Neuroinflammation peak time	CT1
	*Significance in* amplitude difference	NA
B. *Per1*	Ctrl is rhythmic.	Y (2.19E-03)
	Neuroinflammation is rhythmic.	N
	*Significance* in phase difference	NA
	Phase difference estimate	NA
	Ctrl peak time	CT15
	Neuroinflammation peak time	NA
	*Significance in* amplitude difference	NA
C. *Ccl3*	Ctrl is rhythmic.	N
	Neuroinflammation is rhythmic.	N
C. *Ccl5*	Ctrl is rhythmic.	Y (2.19E-03)
	Neuroinflammation is rhythmic.	N
	*Significance* in phase difference	NA
	Phase difference estimate	NA
	Ctrl peak time	CT0
	Neuroinflammation peak time	NA
	*Significance in* amplitude difference	NA

Statistical analysis of rhythmicity and intergroup comparisons corresponding to **Figure 4** using the R package CircaCompare. Rhythmicity and differences are considered significant when the P value is < 0.05. Y, yes; N, no; NA, not available. Period length in hour (h).

To investigate the potential mechanism, we analyzed core clock components. *Bmal1* expression was phase-shifted in neuroinflammatory microglia, and its downstream target *Per1* lost rhythmicity (**Figure 4C**; **Table 3B**). These findings indicate that the intrinsic microglial clocks are disrupted during neuroinflammation and may contribute to persistently enhanced microglial activation marker expression.

Under neuroinflammatory conditions, microglia secrete various cytokines and chemokines to promote pro-inflammatory reactions (1, 53, 54). To investigate whether microglial clocks modulate cytokine expression, we analyzed protein levels of secreted cytokines and chemokines in purified microglia. Under neuroinflammatory conditions, several factors, including CC chemokine ligands CCL3 and CCL5, were upregulated (**Supplementary Figure S2A**). Gene expression analysis revealed that *Ccl5* was rhythmic under control conditions but lost rhythmicity during neuroinflammation. In contrast, *Ccl3* remained arrhythmic in both conditions, with overall expression induced during neuroinflammation (**Figure 4D**; **Table 2C**). To further assess whether microglial clocks regulate cytokine expression more broadly, we examined additional genes, including *Ccl12* and *Il-1β*, and found that their expression was non-rhythmic even under physiological conditions (**Supplementary Figures S2B, C**). These findings suggest that microglial clocks selectively regulate a subset of immune-related genes, and that this regulation is disrupted during neuroinflammation.

### Microglial clocks promote OPC recruitment under physiological conditions

Disrupted BMAL1 downstream in neuroinflammatory microglia led us to hypothesize that clock dysfunction contributes to the loss of microglial physiological functions. To investigate the role of microglial clocks under physiological conditions, we used two transgenic mouse lines: CX_3_CR-1^GFP^ knock-in mice, which express enhanced green fluorescent proteins (EGFP) in microglia and other myeloid cells (**Supplementary Figure S3**) (41), and NG2^DsRed^ mice, which express red fluorescent protein under the neural/glial antigen 2 (NG2) promoter to label NG2^+^ OPCs (42).

**Figure 5:**
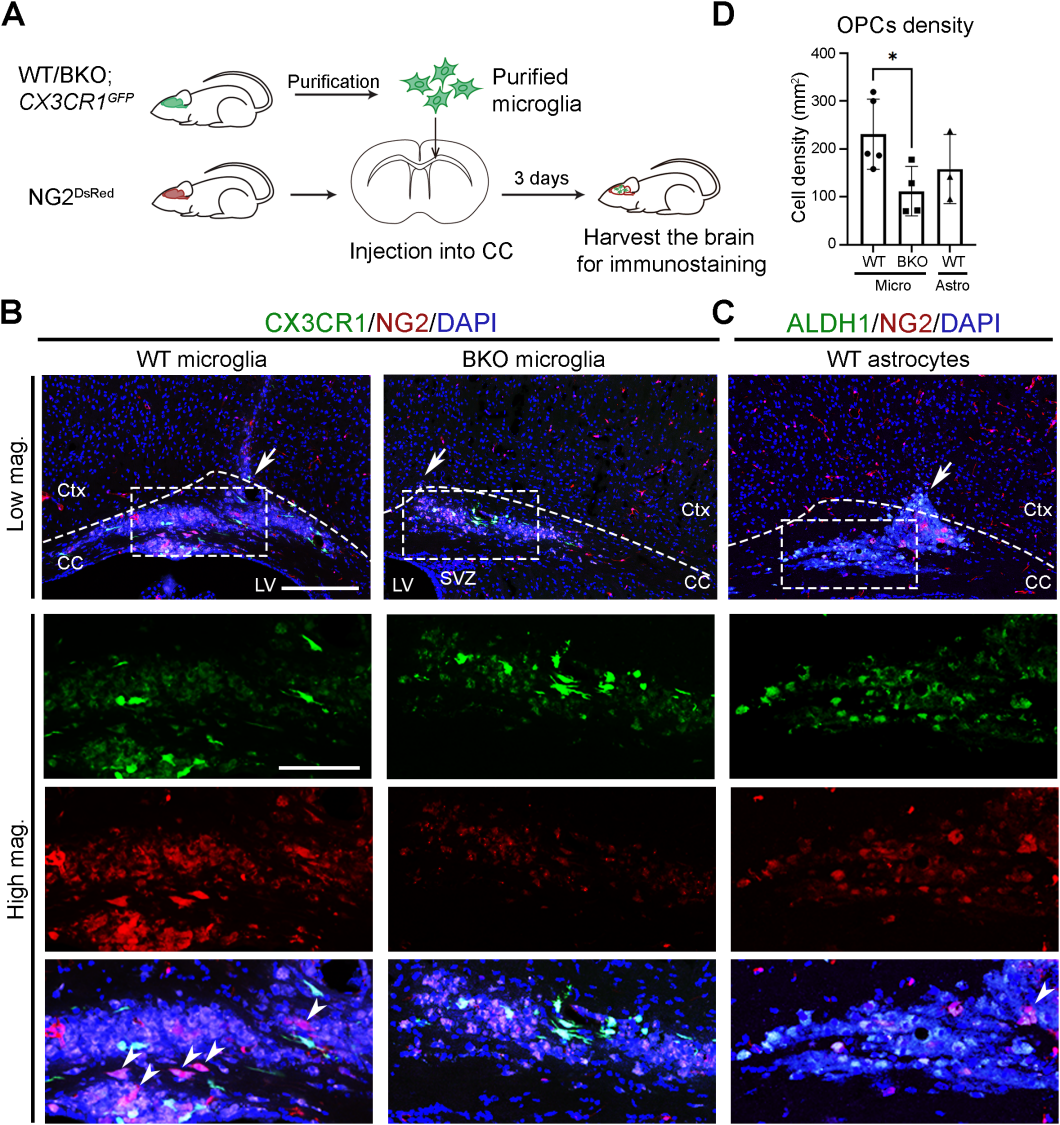
Microglial clocks promote OPC recruitment under physiological conditions. **(A)** Experimental workflow illustrating the preparation of microglia, their injection into the corpus callosum (CC), and subsequent brain harvesting for immunohistochemistry. Donor mice were sacrificed at circadian time (CT) 04, and purified microglia were transplanted into the CC of recipient mouse at CT10. Recipient mice were harvested at CT06 on day 3 post-transplantation. **(B)** Confocal images of the CC and overlying cortex in NG2^DsRed^ mice following injection of GFP-expressing microglia (CX3CR1, green) purified from wild-type (WT) or conventional *Bmal1* knockout (BKO) mice on a CX3CR-1^GFP^ background. NG2&^+^ cells (red) mark oligodendrocyte progenitor cells (OPCs). **(C)** Confocal images of the CC in NG2^DsRed^ mice following injection of GFP-expressing astrocytes (ALDH1L1, green) purified from WT on an Aldh1l1-EGFP background. DAPI (blue) serves as a nuclear counterstain. Dashed boxes in lower magnification (Low mag.) images indicate regions enlarged in higher magnification (High mag.) panels. Dashed line outlines the cortex (Ctx) and CC. White arrows in the Low mag. images mark the injection sites. White arrowheads indicate cells expressing cytoplasmic NG2. Images are maximum intensity projections of three z-stacks. Scale bars (white bars in the first panel): 200 μm (Low mag.), 50 μm (High mag.). **(D)** Quantification of NG2&^+^ cell density at the injection site from **(B, C)**, presented as mean ± SD. n ≥ 3 mice per group. Each dot represents one mouse. Asterisk indicates statistical significance (Student’s t-test). *P value < 0.05.

Under physiological conditions, microglia support oligodendrocyte lineage cells by secreting trophic factors such as insulin-like grow factor 1 (IGF-1) and transforming growth factor β (TGF-β), which promote OPC recruitment, proliferation, and differentiation (55–58). These OPCs replace senescent or damaged oligodendrocytes and contribute to spontaneous myelin turnover in the brain (59). To initiate repair, OPCs first migrate to affected areas and then differentiate into mature oligodendrocytes that re-form myelin around axons (60–62). To test whether microglial clocks contribute to OPC recruitment, we crossed CX_3_CR-1^GFP^ mice with conventional *Bmal1* knockout (BKO) mice to generate CX_3_CR-1^GFP:^ BKO line. Purified GFP-expressing microglia from CX_3_CR-1^GFP^ (mWT) and CX_3_CR-1^GFP:^ BKO (mBKO) mice were transplanted into the corpus callosum (CC) of NG2^DsRed^ mice (**Figure 5A**). The CC provides an ideal site for OPC analysis due to its high density of parenchymal OPCs and aligned myelinated axons that serve as migration tracks. Three days after transplantation, significantly more NG2&^+^ cells were detected at mWT injection sites compared to mBKO (**Figures 5B, D**). In the mBKO-injected CC, some red fluorescence overlapped with nuclear counterstain, indicating non-specific signals, as NG2 is a cytoplasmic marker. These results suggest that functional microglial clocks are required for effective OPC recruitment.

To determine whether this increase was due to OPC migration rather than proliferation, we co-stained NG2&^+^ cells with the proliferation marker Ki67. Most NG2&^+^ cells were Ki67–negative at both the injection site (#1) and surrounding areas (#2) (**Supplementary Figure S4**), indicating that microglial clocks primarily influence recruitment rather than local OPC proliferation.

To test whether this effect is specific to microglia, we transplanted purified GFP-expressing astrocytes from Aldh1l1-EGFP mice (**Supplementary Figure S5**) into the CC of NG2^DsRed^ mice. In contrast to mWT microglia, astrocyte transplantation did not increase NG2&^+^ cell numbers compared to mBKO-injected sites (**Figures 5C, D**). These results indicate a microglia-specific, clock-dependent mechanism underlying OPC recruitment under physiological conditions.

### Neuroinflammatory microglia lose their capacity to recruit OPCs

**Figure 6:**
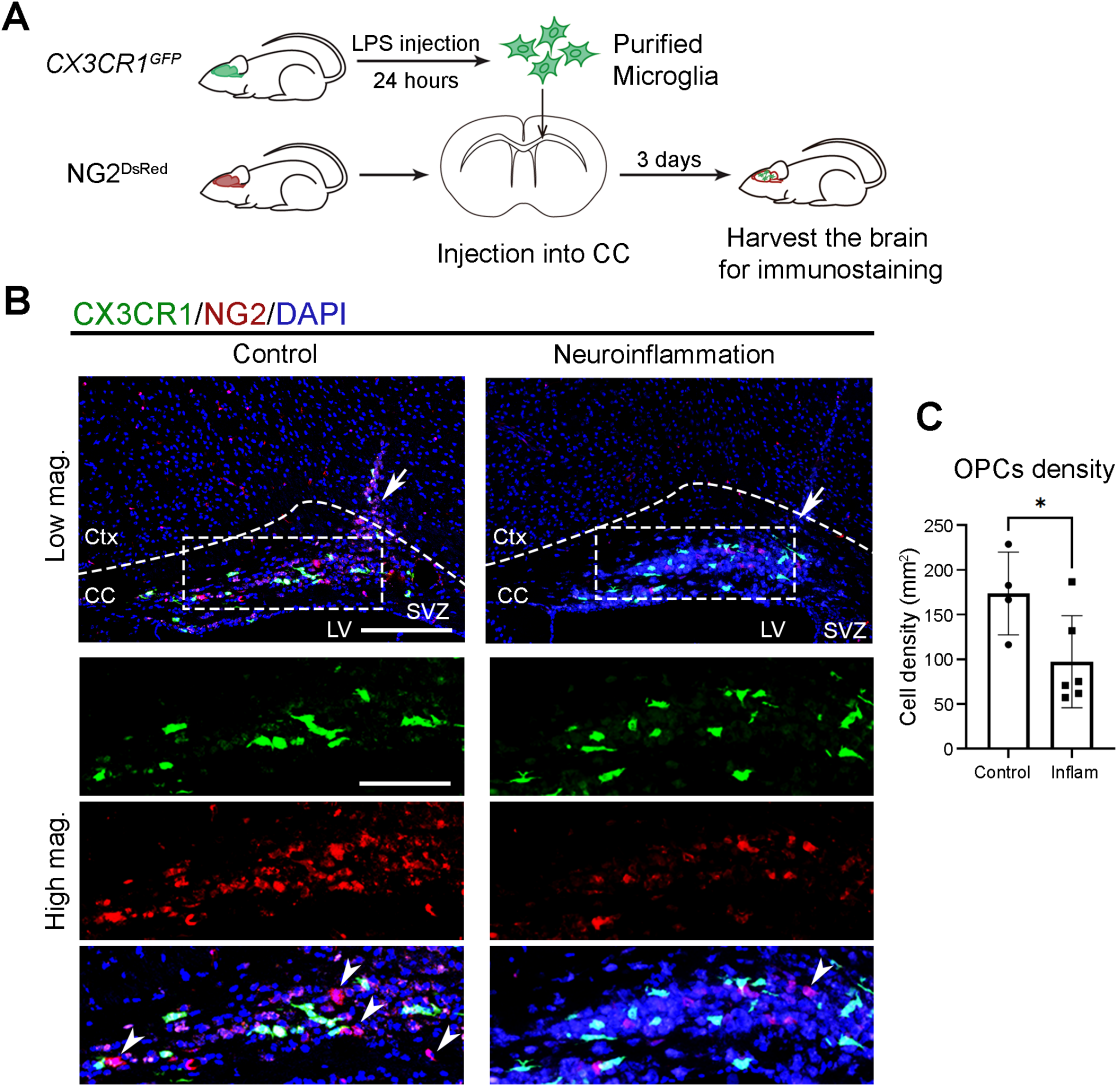
Neuroinflammatory microglia lose their capacity to recruit OPCs. **(A)** Experimental workflow illustrating the induction of neuroinflammation, microglial isolation, local injection into the corpus callosum (CC), and subsequent brain harvesting for immunostaining. Donor mice received either PBS (control) or lipopolysaccharide (LPS) via intraperitoneal injection and were sacrificed at circadian time (CT) 04, 24 hours post-injection. Purified microglia were then transplanted into the CC of recipient mouse at CT10. Recipient mice were harvested at CT06 on day 3 post-transplantation. **(B)** Confocal images of the CC and overlying cortex in NG2^DsRed^ mice following injection of GFP-expressing microglia (CX3CR1, green) purified from PBS-injected control or LPS-injected neuroinflammatory CX3CR-1^GFP^ mice. NG2&^+^ cells (red) mark oligodendrocyte progenitor cells (OPCs). DAPI (blue) serves as a nuclear counterstain. Dashed boxes in lower magnification (Low mag.) images indicate regions enlarged in higher magnification (High mag.) panels. Dashed line outlines the cortex (Ctx) and CC. White arrows in the Low mag. images mark the injection sites. White arrowheads indicate cells expressing cytoplasmic NG2. Images are maximum intensity projections of three z-stacks. Scale bars (white bars in the first panels): 200 μm (Low mag.), 50 μm (High mag.). **(C)** Quantification of NG2&^+^ cell density at the injection site from **(B)**, presented as mean ± SD. Each dot represents one mouse. n ≥ 4 mice per group. Asterisk indicates statistical significance (Student’s t-test). *P value < 0.05. LPS, lipopolysaccharide.

To test whether neuroinflammatory conditions impair the clock-dependent function of microglia in supporting OPC recruitment, we injected purified microglia from control and neuroinflammatory CX_3_CR-1^GFP^ mice into the CC of NG2^DsRed^ mice (**Figure 6A**). Three days after transplantation, significantly fewer NG2&^+^ cells were observed at sited injected with neuroinflammatory microglia compared to control microglia (**Figures 6B, C**). This result suggests that, under neuroinflammatory conditions, microglia shift their functional priority from physiological roles, such as OPC recruitment, toward immune responses.

In summary, our study demonstrates that functional microglial clocks are essential for supporting OPC recruitment under physiological conditions. During neuroinflammation, however, *Bmal1* expression in microglia undergoes a phase shift, and its target gene *Per1* – as well as microglial activation markers *Iba1* and *Itgam* – loses rhythmicity while their expression levels increase. This disruption in circadian clock output is accompanied by sustained cytokine production and loss of rhythmic regulation, leading to a persistently activated microglia state in neuroinflammation. These findings highlight the importance of intact microglial clocks in maintaining physiological roles and suggest that preserving circadian clock function may help mitigate excessive neuroinflammatory responses while supporting beneficial microglial activity.

## Discussion

Microglial circadian clocks have received less attention than those of neurons and astrocytes. Most previous studies have largely focused on the observation that microglial responses to immune stimuli, such as LPS, vary depending on time-of-day (36), rather than directly addressing microglial clock function under physiological and inflammatory conditions. More recent evidence demonstrates that microglia contain autonomous molecular clocks that generate daily oscillations in physiological and transcriptional activity, including the rhythmic production of cytokines (20, 22). These oscillations are thought to modulate time-of-day-dependent immune responses to challenges. However, how endogenous microglial clocks respond to neuroinflammatory conditions and contribute to functional interactions with other brain cells have not been clearly defined.

In this study, we first confirmed that the presence of cell-autonomous clocks in microglia by monitoring circadian PER2::Luc bioluminescence rhythms in primary microglial cultures. This provided the foundation for investigating microglial clock function *in vivo* under physiological conditions. Through microglial transplantation experiments, we demonstrated that wild-type microglia, but not microglia with disrupted clocks, recruit NG2&^+^ OPCs under physiological conditions. Microglia are known to maintain brain homeostasis and promote tissue repair through clearance of cellular debris and release of trophic factors (9, 53). Our findings support that microglial clocks regulate these supportive functions, such as facilitating OPC recruitment for oligodendrocyte replacement.

Under physiological conditions, our study specifically focused on the recruitment stage of NG2&^+^ OPCs, rather than their subsequent differentiation or maturation into myelinating oligodendrocytes. This focus was guided by biological rationale and technical limitations. NG2 is a well-established OPC marker that is rapidly downregulated upon differentiation. Since the CC is densely populated with mature oligodendrocytes and myelinated axons, microglia-recruited NG2&^+^ cells that undergo differentiation would lose NG2 expression and become indistinguishable from pre-existing oligodendrocytes. By examining NG2&^+^ cells three days after microglial transplantation, we minimized interpretive ambiguity and focused our analysis on the OPC recruitment phase.

To explore how neuroinflammation affects microglial clocks and their function, we used a systemic LPS injection model. Although LPS is administered peripherally, its robust and reproducible induction of microglial activation in the brain is well established and widely used as a neuroinflammation model (63, 64). Although our microglia isolation does not distinguish between resident microglia and infiltrating monocyte-derived macrophages, the analysis time point (three days post-LPS injection) supports our interpretation that the majority of cells analyzed were microglia. Previous studies conducted at similar time points and LPS doses showed that resident microglia still remain the dominant myeloid population in the parenchyma at this time point (65, 66). These studies indicate that significant monocyte infiltration typically occurs at later time points or under more severe or chronic inflammation. However, we recognize the potential for partial monocyte contribution and have taken this into consideration in interpreting our findings and future studies.

Rhythmic gene expression analysis showed that microglial activation markers *Iba1* and *Itgam* oscillate under physiological conditions. However, their rhythmicity is phase-shifted or lost under neuroinflammatory conditions, coinciding with altered expression patterns of core clock genes *Bmal1* and *Per1*. Although *Bmal1* expression remains detectable, it is phase-shifted, suggesting that altered circadian timing, rather than reduced expression, can disrupt rhythmic output. *Per1*, a well-known BMAL1 target, becomes arrhythmic under neuroinflammation, further supporting the interpretation that clock-controlled output pathways are disrupted. These changes are unlikely to result from direct transcriptional regulation of *Iba1* and *Itgam* by BMAL1. Instead, these disruptions may reflect indirect effects mediated by downstream clock-regulated transcriptional networks. Previous studies support this interpretation, showing that many immune-related genes are regulated indirectly by circadian clocks (67, 68). Together, these findings suggest that microglial clocks generate the rhythmicity in the expression of microglial activation markers, rather than controlling their absolute expression levels.

Our data do not suggest that circadian clock disruption directly causes microglial activation. Under physiological conditions, microglia with functional clocks exhibit circadian oscillations in activation marker expression. These observations indicate that microglia can enter active states while maintaining intact clocks. We propose that microglial clocks regulate the timing of activation rather than the presence or absence of activation itself. Once microglia respond to inflammatory stimuli and become activated, this rhythmic regulation is lost due to disruption of the underlying molecular clocks. This interpretation implies that overexpressing *Bmal1* alone may not restore rhythmic function if the introduced construct does not oscillate. Restoring circadian rhythmicity—not merely increasing expression—appears necessary to recover clock-dependent microglial functions.

We also investigated candidate immune genes under physiological conditions and found that only *Ccl5* exhibited circadian rhythmicity, but *Ccl3*, *Ccl12*, and *Il-1β* did not. These findings support the idea that circadian clocks selectively regulate a subset of immune-related genes. Microglia produce a broad range of cytokines, chemokines, and trophic factors even in the absence of inflammation (**Supplementary Figure S2A**). Therefore, we did not limit our analysis to a small number of pre-selected candidates, as this would provide only partial insight into the underlying mechanisms. Instead, we emphasized the *in vivo* functional consequences of microglial clock activity. However, identifying the specific molecular mediators responsible for OPC recruitment remains an important direction for future investigation.

In conclusion, our findings complement growing evidence that circadian clocks across glial cell types contribute to brain homeostasis. Astrocytes, for example, contain intrinsic circadian oscillators that regulate their physiology and influence neuronal timing and immune responses (69, 70). To move forward, the coordination between glial clocks should be further explored to understand how intercellular circadian alignment is maintained and disrupted in pathological conditions, and whether restoring microglial clock function can modulate glial interactions and support recovery.

## Data Availability

The original contributions presented in the study are included in the article/**Supplementary Material**. Further inquiries can be directed to the corresponding author.
