# Supplementary material for: Microglial clock dysfunction during neuroinflammation impairs oligodendrocyte progenitor cell recruitment and disrupts neuroimmune homeostasis
Source: Front Immunol. 2025 Jul 7;16:1620343. doi: 10.3389/fimmu.2025.1620343 (PMC12277320; doi:10.3389/fimmu.2025.1620343)
Supplement: Supplementary file 1 [file DataSheet1.pdf]

Supplementary Information

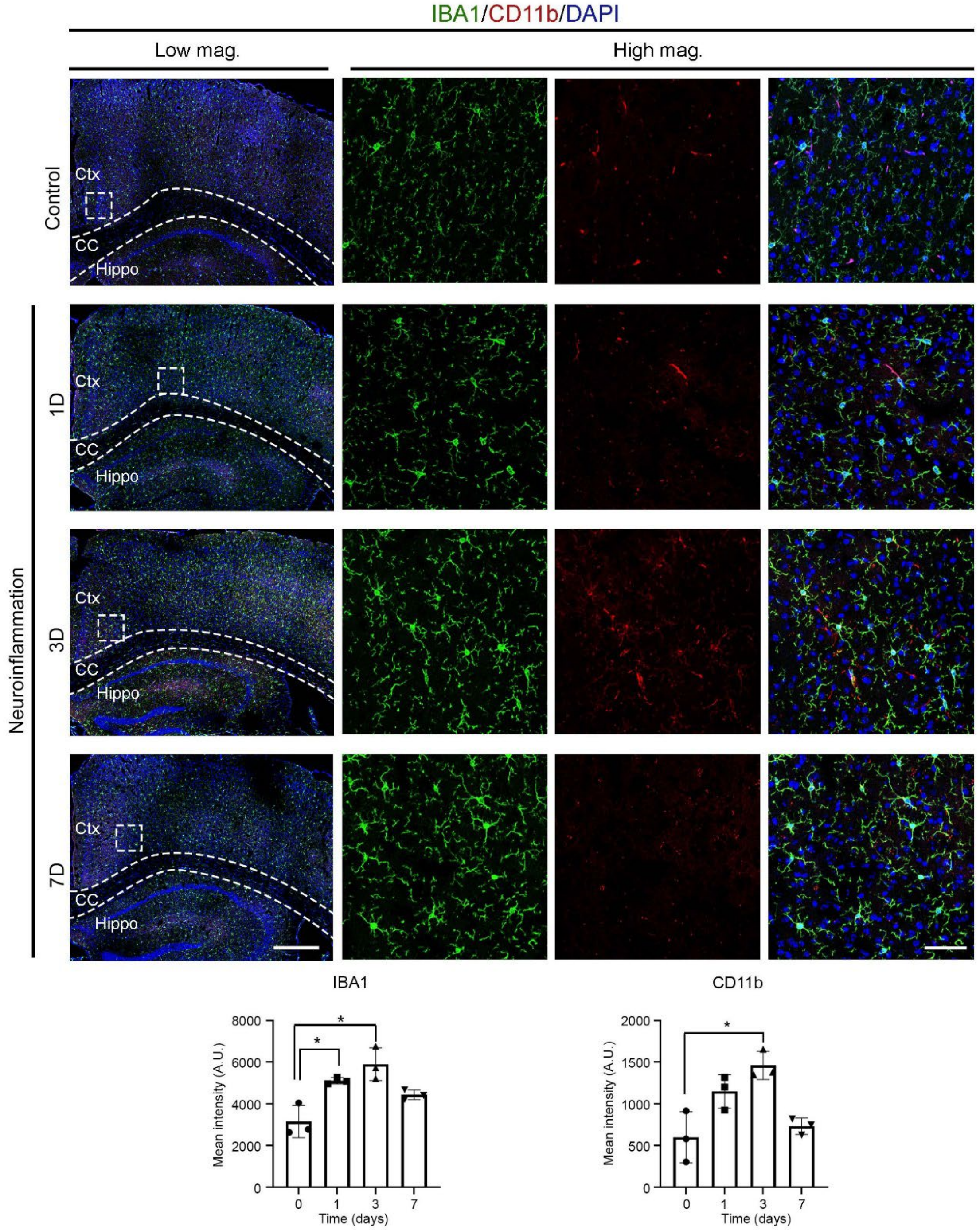

Supplementary Figure S1. Lipopolysaccharide-induced neuroinflammation in the brain. Confocal images of wild-type mouse brain following intraperitoneal injection of either PBS (control) or lipopolysaccharide (LPS; neuroinflammation) at circadian time (CT) 04. Brains were harvested at CT06 on Day 1 (1D), Day 3 (3D), and Day 7 (7D) post-injection and stained with antibodies against IBA1 (green) and CD11b (red). DAPI (blue) serves as a nuclear counterstain. Dashed boxes in lower magnification (Low mag.)

images indicate regions enlarged in higher magnification (High mag.) panels. Dashed lines delineate the cortex (Ctx), corpus callosum (CC), and hippocampus (Hippo). Images are maximum intensity projections of three z-sections. Scale bars (white bars in bottom panels): 200 µm (Low mag.), 50 µm (High mag.). Quantification is shown in the bar graph, presented as mean ± SD. n = 3 mice per time point. Each dot represents one mouse. X-axis indicates days post-injection. Asterisks denote statistical significance (Student's t-test). *, P value < 0.05.

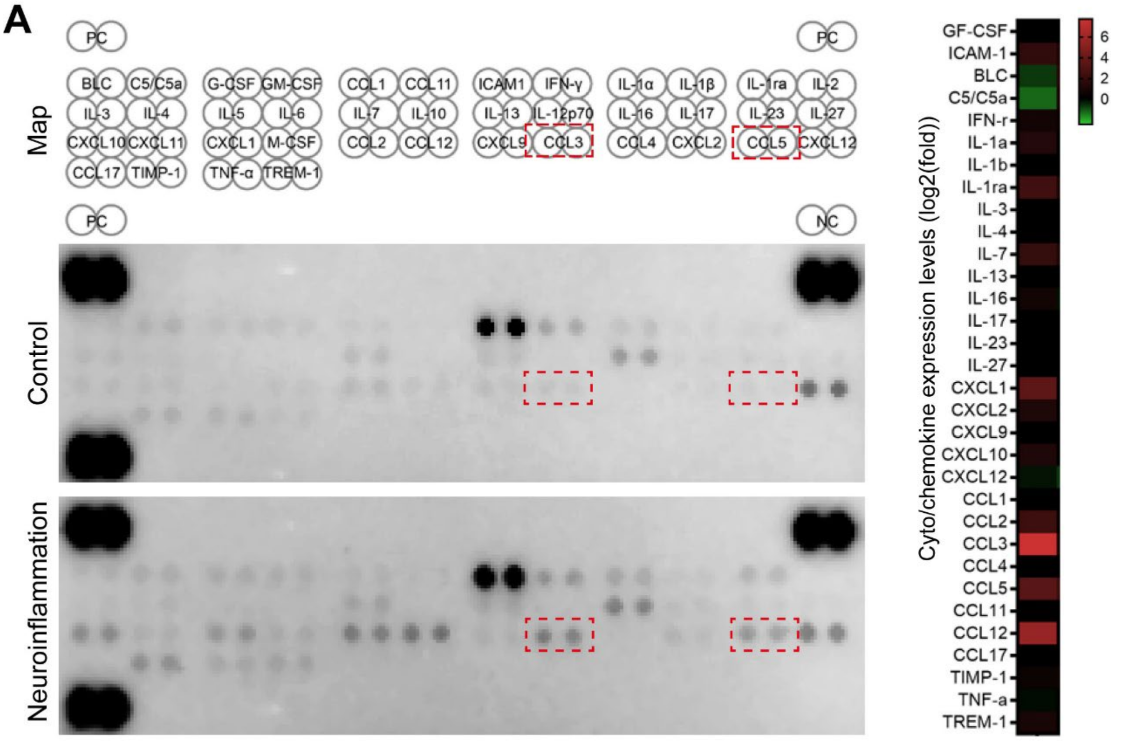

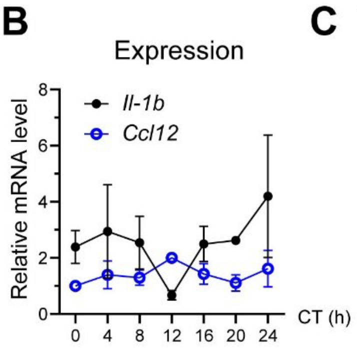

C

Parameter	Method	Rhythmic or not (Y/N)	*P* value or rhythmic	Period length (h)
Il-1b	JTK	N	4.75E-01	NA
	LS	N	6.41E-01	NA
	Meta_2d	N	6.67E-01	NA
Ccl12	JTK	N	1.00E00	NA
	LS	N	9.99E-01	NA
	Meta_2d	N	1.00E00	NA

Supplementary Figure S2. Cytokines and chemokines produced by control and neuroinflammatory microglia. (**A**) Cytokine and chemokine assays performed on protein extracts from microglia purified from control and neuroinflammatory mice. Mice received an intraperitoneal injection of either PBS (control) or lipopolysaccharide (LPS, neuroinflammation) at circadian time (CT) 04. Brains were harvested three days post-injection at CT04 for microglia purification. Top panel indicates the position of each cytokine and chemokine on the membrane. The heatmap shows quantification normalized to the control condition. (**B**) Gene expression of *Il-1β* (black) and *Ccl12* (blue) in control microglia across the circadian cycle, measured by qRT-PCR. Expression values are normalized to a control gene (*Rps13*) and presented as mean ±

SD. n = 3 mice at each CT. (**C**) Statistical analysis of *Il-1β* and *Ccl12* rhythmicity using the R package MetaCycle, which integrates three independent methods: JTK_CYCLE (JTK), Lomb-Scargle (LS), and ARER (Meta_2d). Rhythmicity was considered significant when the P value is < 0.05. Y, yes, rhythmic; N, no, non-rhythmic; NA, not available. Period length is in hour (h).

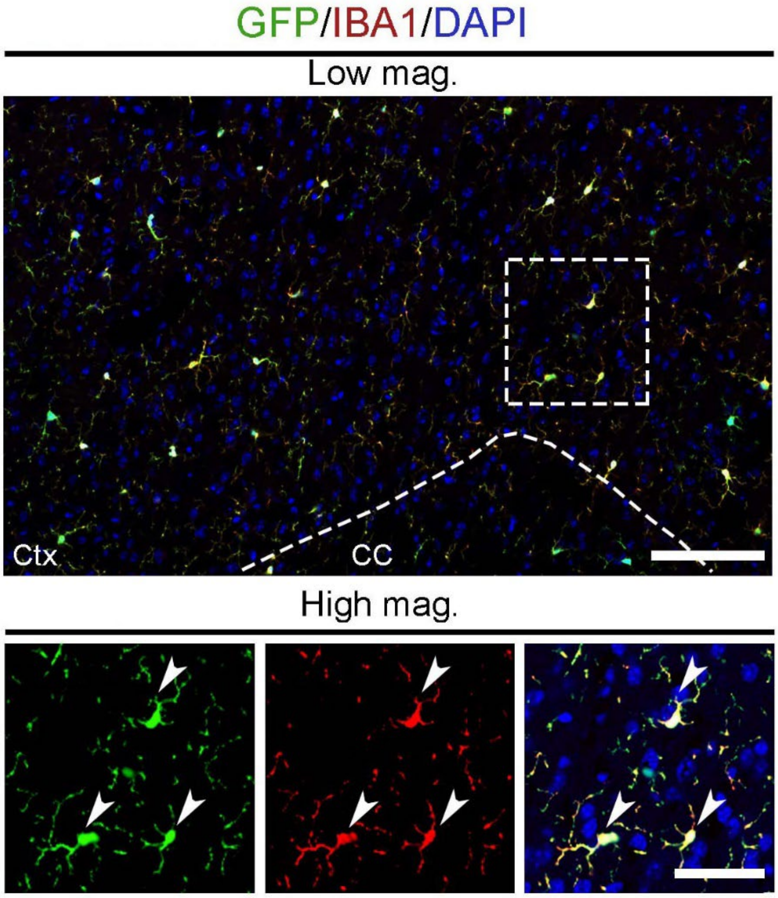

Supplementary Figure S3. GFP-expressing microglia in the brain of CX3CR1^{GFP} mice. Confocal images of the cortex (Ctx) in CX3CR1^{GFP} mice harvested at circadian time 06, stained with anti-IBA1 antibodies (red). GFP^{+} cells (green) represent microglia. DAPI (blue) serves as a nuclear counterstain. n = 1. Dashed box in lower magnification (Low mag.) image indicates a region shown at higher magnification (High mag.) panels. Dashed line outlines the Ctx and corpus callosum (CC). White arrowheads indicate cells double-positive for GFP and IBA1. Images are maximum intensity projections from three confocal z-planes. Scale bars (white bars in panels): 100 µm (Low mag.), 50 µm (High mag.).

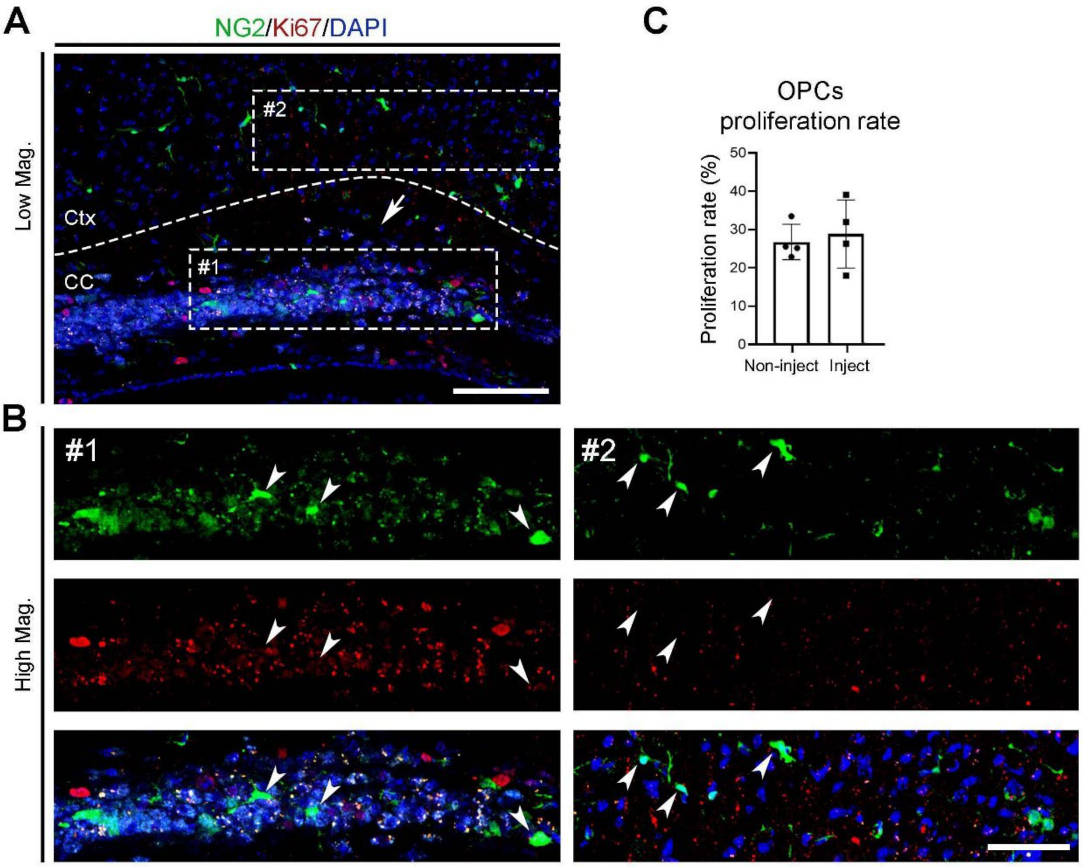

Supplementary Figure S4. No significant effect of microglia on OPC proliferation under physiological conditions. (**A**-**B**) Confocal images of the brain in $NG2^{DsRed}$ mice following the injection of microglia purified from wild-type control mice on a $CX3CR1^{GFP}$ background. Sections were stained with antibodies against Ki67 (red), a marker of cell proliferation. $NG2^{+}$ cells (green) mark oligodendrocyte progenitor cells (OPCs). DAPI (blue) serves as a nuclear counterstain. (**A**) Sites labeled #1 and #2 indicate the injection site and adjacent area, respectively. Dashed boxes in low-magnification (**A**; Low mag.) images indicate regions shown at higher magnification (**B**; High mag.). Dashed line outlines the cortex (Ctx) and corpus callosum (CC). White arrow in the Low mag. image marks the injection site. White arrowheads highlight $NG2^{+}$ cells but Ki67 signal negative. Images are maximum intensity projections of three z-stacks. Scale bars (white bars in panels): 100 µm (Low mag.), 50 µm (High mag.). (**C**) Quantification of $NG2^{+}/Ki67^{+}$ cell numbers from (B), presented as mean ± SD. n ≥ 3 mice per group. Each dot represents one mouse.

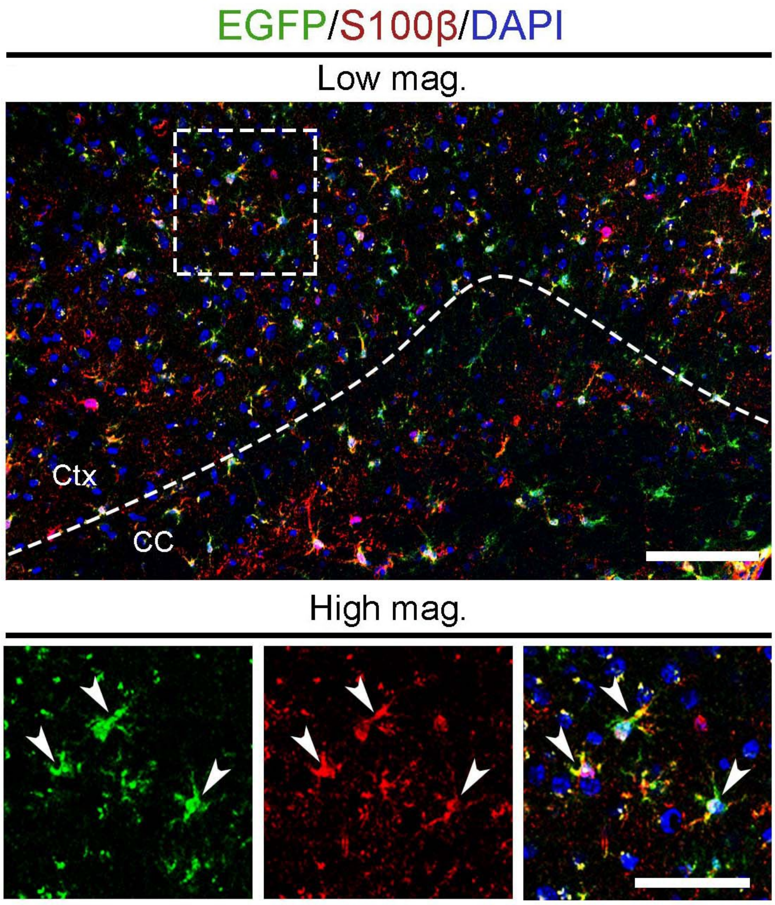

Supplementary Figure S5. GFP-expressing astrocytes in the brain of Aldh1l1-EGFP mice. Confocal images of the cortex (Ctx) and corpus callosum (CC) in Aldh1l1-EGFP mice harvested at circadian time 06, stained with antibodies against S100β (red). $EGFP^+$ cells (green) represent astrocytes. DAPI (blue) serves as a nuclear counterstain. $n = 1$. Dashed box in lower magnification (Low mag.) image indicates a region shown at higher magnification (High mag.) panels. Dashed line outlines the Ctx and CC. White arrowheads indicate astrocytes that are double-positive for EGFP and S100β. Images are maximum intensity projections of three z-stacks. Scale bars (white bars in panels): 100 µm (Low mag.), 50 µm (High mag.).
